# USP52 inhibits cell proliferation by stabilizing PTEN protein in non-small cell lung cancer

**DOI:** 10.1042/BSR20210486

**Published:** 2021-10-04

**Authors:** Maoshu Zhu, Hui Zhang, Fuhua Lu, Zhaowei Wang, Yulong Wu, Huoshu Chen, Xin Fan, Zhijiang Yin, Fulong Liang

**Affiliations:** 1 Research Department, The Fifth Hospital of Xiamen, Xiamen, 361101, China; 2 Xiang’an Branch, The First Affiliated Hospital of Xiamen University, 3611101, China; 3 Internal Medicine Department, The Fifth Hospital of Xiamen, Xiamen, 361101, China; 4 Gynecology Department, The Fifth Hospital of Xiamen, Xiamen, 361101, China; 5 Surgery Department, The Fifth Hospital of Xiamen, Xiamen, 361101, China; 6 Pharmacy Department, The Fifth Hospital of Xiamen, Xiamen, 361101, China; 7 Oncology Department, Xiamen Haicang Hospital, Xiamen 361026, China

**Keywords:** cell proliferation, inhibition, Non-small cell lung cancer, PTEN, stabilization, USP52

## Abstract

Non-small cell lung cancer (NSCLC) is the most common subtype of lung cancer. Ubiquitination is closely related to the development of lung cancer. However, the biological importance of newly discovered ubiquitin-specific peptidase (USP) 52 (USP52) in NSCLC remained unclear. Here, our findings identify USP52 as a novel tumor suppressor of NSCLC, the low expression of USP52 predicts a poor prognosis for NSCLC patients. The present study demonstrates that USP52 inhibits cancer cell proliferation through down-regulation of cyclin D1 (CCND1) as well as AKT/mTOR signaling pathway inhibition. Meanwhile, USP25 also suppresses NSCLC progression via enhancing phosphatase and tensin homolog (PTEN) stability in cancer cells, which further indicates the significance/importance of USP52 in NSCLC suppression.

## Introduction

Lung cancer is a leading cause of cancer-related deaths worldwide. Non-small cell lung cancer (NSCLC) is the most frequent subtype of lung cancer [1]. In recent years, great progress has been made in the research of lung cancer. The understanding of genetic alterations that drive NSCLC is evolving. Nonetheless, the prognosis of NSCLC remains poor owing to the lack of effective treatment [2].

Ubiquitin proteases are a large family of cysteine proteases that specialize in cleavage of ubiquitin conjugates [3]. The ubiquitin system regulates essential cellular processes in eukaryotes. Ubiquitin binds to substrate proteins in a monomeric or chain formation/structure, and the ubiquitin-modified topological structure regulates the interactions between substrate and specific proteins. Therefore, ubiquitination directs the fate of many substrates, including proteasome degradation. The deubiquitinases drive the process of ubiquitin cleavage from substrates [4]. This process controls the stability of most cellular proteins, and its abnormal regulation leads to the occurrence of various human diseases, including cancer. Deubiquitinase inhibition can induce the degradation of selected proteins, which potentially includes some otherwise undruggable targets. For instance, the inhibition of ubiquitin-specific protease (USP) 7 (USP7) results in the degradation of the oncogenic E3 ligase MDM2 and the reactivation of the tumor suppressor p53 in various cancers [5].

The USP52, also called poly(A)-specific ribonuclease subunit PAN2, was reported as a key component of P-bodies required to prevent HIF1α mRNA degradation [6]. It was also reported that USP52 plays an important role in inhibiting antiviral immunity [7]. Additionally, USP52 could promote histone chaperone ASF1A stabilization [8]. However, the antitumor function of USP52 has not been reported.

Rapid proliferation is a malignant phenotype of NSCLC. The proliferation of cancer cells is regulated by a variety of proteins and genes. Long noncoding RNA PVT1 promotes NSCLC cell proliferation through epigenetically regulating LATS2 expression [9]. IL-6 induces neuroendocrine dedifferentiation and cell proliferation in NSCLC cells [10]. Prognostic significance of cyclin D1 (CCND1) as a cell cycle promoter can be overexpressed in primary resected NSCLC [11]. The expression level of cyclin-dependent kinase (CDK) 2 (CDK2) can also be elevated in NSCLC for the enhancement of cancer cell proliferation [12,13].

It is well known that phosphatase and tensin homolog (PTEN) is an important tumor suppressor in NSCLC. The PTEN/phosphatidylinositol 3-kinase (PI3K)/AKT pathway regulates multiple cellular functions, including cell growth, proliferation, survival, motility, differentiation, invasion and intracellular trafficking [14]. Alterations in this pathway, particularly PTEN inactivation, have been associated with resistance to the therapy of EGFR-tyrosine kinase inhibitor and results in lower survival in NSCLC patients [15].

PTEN, as another commonly mutated, deleted and epigenetically silenced tumor suppressor in human lung cancers [16], is altered in 15% of human lung small cell cancers (SCCs) [17]. PTEN negatively regulates the PI3K/AKT pathway, which is also altered in more than half of human NSCLCs [17]. Here, we used human NSCLC and cell lines to research the expression of USP52, and the regulatory relationship between USP52 and PTEN. We discovered that USP52 plays an important role in NSCLC suppression by inhibiting cancer cell proliferation via PTEN stabilization, which further indicated that USP52 plays an important role in NSCLC suppression. Thus, the low expression of USP52 predicts a poor prognosis for NSCLC patients.

## Materials and methods

### Survival analysis

*USP52* gene expression profile (USP52 data) for lung cancer patients was obtained from TCGA data portal (https://tcga-data.nci.nih.gov/tcga/). Clinical data such as gender, age, histological type, survival and outcome were also downloaded from TCGA data portal.

### Molecular biology

HA-tagged USP52 constructs were made using the pCDH-GFP-Puro vector (https://www.addgene.org/167463/). Sequences encoding the HA epitope were added by PCR through replacement of the first Met-encoding codon in the respective cDNA clones.

To produce USP52-overexpressing cell lines, H292 and H460 cells were infected with lentivirus particles expressing HA-USP52 plasmids in the presence of 10 µg/ml polybrene. Cells infected with retroviruses expressing pCDH-GFP-Puro plasmids were used as controls.

### Cell culture and treatments

Human bronchial epithelial cells (HBE), NSCLC cell lines H292, H460 and LTEP-A-2 were obtained from American Type Culture Collection (ATCC; Manassas, VA, U.S.A.). Cells were cultured in a medium containing 10% fetal bovine serum (FBS; HyClone Laboratories Inc., Logan, UT, U.S.A.) and 1% penicillin–streptomycin (Invitrogen, Carlsbad, CA, U.S.A.) at 37°C in the presence of 5% CO_2_. Media containing cells were passaged every 2–3 days. Four independent series of treatments were conducted to obtain the three technical repeats that were used for all studies.

### Study of human primary lung cancer specimens

The present study was approved by the Medical Ethics Committee of The Fifth Hospital of Xiamen, and patients provided written informed consent in accordance with the legal and institutional ethical guidelines defined by the hospital (ethical approval number: 2019-XMSDWYYLL-051). We collected 18 lung cancer specimens with tumor-adjacent lung tissues from patients who had undergone resection at the Fifth Hospital of Xiamen and stored the tissues at the tissue bank of the Fifth Hospital of Xiamen. The patients were diagnosed with NSCLC at the Fifth Hospital of Xiamen, and diagnoses were made according to the World Health Organization criteria.

### RNA extraction and quantitative reverse transcription polymerase chain reaction

RNA was extracted using TRIzol reagent (Invitrogen, Carlsbad, CA, U.S.A.). Complementary DNA was synthesized using Invitrogen SuperScript III Reverse Transcriptase kit according to the manufacturer’s protocol (Invitrogen, Carlsbad, CA, U.S.A.). Besides, SYBR Green-based RT-qPCR was performed using Mx3000P qPCR System (Stratagene California, San Diego, CA, U.S.A.) according to the manufacturer’s instructions. Three independent amplifications were performed for each sample in each technique. In addition, GAPDH was taken as an internal reference into consideration. The primer sequences used for quantitative reverse transcription polymerase chain reaction (RT-qPCR) are shown in Table 1.

**Table 1:** Primer sequences

Name	Accession numbers	Forward sequence	Reverse sequence	Amplicon size
*USP52*	NM_001394701	GTGGGTGTACCTGTTTCCGT	GGATGAGTAGCGCTCCAAGG	117
*GAPDH*	NM_001256799	AAGCCTGCCGGTGACTAAC	GTTAAAAGCAGCCCTGGTGAC	174
*PTEN*	NM_000314	TCCCAGACATGACAGCCATC	TGCTTTGAATCCAAAAACCTTACT	190

### Cell proliferation analysis

According to the kit manual instructions, cell proliferation assay was performed using the CCK-8. The transfected cells (5 × 10^3^) were seeded in 96-well plates with 90 µl medium containing 10% FBS in each well. After different treatments, 10 μl CCK-8 solution was added to each well with 100 μl culture medium following 2-h incubation at 37°C, the number of cells in each well was calculated by measuring the absorbance at 450 nm using Microplate Reader.

### Western blot analysis

Protein extracts were prepared by lysis in a buffer containing 50 mM Tris-HCl (pH 7.4), 0.1% Triton X-100, 5 mM EDTA, 250 mM NaCl, 50 mM NaF, 0.1 mM Na_3_VO_4_ and protease inhibitors (Roche, Basel, Switzerland). Proteins were separated by SDS/PAGE, and then transferred on to polyvinylidene difluoride (PVDF) membranes. Blots were probed with the following antibodies: anti-USP52 mouse monoclonal (Santa Cruz Biotechnology, Inc., Dallas, TX, U.S.A., 1:1000), anti-GAPDH mouse monoclonal (Invitrogen, Carlsbad, CA, U.S.A., 1:3000), anti-cyclinD1 mouse monoclonal (Invitrogen, Carlsbad, CA, U.S.A., 1:1000), anti-CDK2 mouse monoclonal (Invitrogen, Carlsbad, CA, U.S.A., 1:1000), anti-p53 mouse monoclonal (Invitrogen, Carlsbad, CA, U.S.A., 1:1000), anti-HA mouse monoclonal (Invitrogen, Carlsbad, CA, U.S.A., 1:2000), anti-p-AKT mouse monoclonal (Invitrogen, Carlsbad, CA, U.S.A., 1:1000), anti-AKT mouse monoclonal (Invitrogen, Carlsbad, CA, U.S.A., 1:1000), anti-p-mTOR mouse monoclonal (Invitrogen, Carlsbad, CA, U.S.A., 1:1000), anti-mTOR mouse monoclonal (Invitrogen, Carlsbad, CA, U.S.A., 1:1000) and mouse monoclonal anti-PTEN antibody (Cell Signaling Technology, Inc., Danvers, MA, U.S.A., 1:1000).

### Immunohistochemical analysis

All specimens were formalin-fixed and paraffin-embedded. Immunohistochemistry (IHC) staining was performed. Briefly, tissue sections were dehydrated in gradient concentration of ethanol solution and fixed with 4% paraformaldehyde at room temperature for 30 min, and then paraffin-embedded. Consecutive 4-µm-sections were used for analysis. Antigen retrieval was performed by microwaving sections in citrate buffer (pH 6.0). Subsequently, slides were incubated overnight with USP52 antibody (Santa Cruz Biotechnology, Inc., Dallas, TX, U.S.A., 1:100) at 4°C. Subsequently, slides were blocked with goat serum (cat. no. AR0009; Wuhan Boster Biological Technology, Ltd; 1:20) at room temperature for 1 h and then incubated with goat anti-rabbit second antibody (cat. no. ab6721; Abcam; 1:2000) at RT for 1 h. Streptococcal avidin-biotinylated peroxidase system (Thermo Fisher Scientific, Inc.) was used to develop the color according to the manufacturer’s instructions. Tissue sections were visualized using an Olympus microscope IX50 at 20× magnification and the images were analyzed using ImageJ 1.49 version software (National Institutes of Health). The American Joint Committee on Cancer (AJCC) Staging Manual system was used to assess tumor grades.

### Protein degradation experiment

H292 cell lines were secondly transfected with control or USP52-HA, respectively, and then, were treated with cycloheximide (CHX) or MG-132 for indicated time periods. After 48 h, cells were harvested, and the expression of PTEN was detected by Western blot analysis and quantification analysis of gray scanning.

### Statistical analysis

All data were presented as mean ± standard deviation (SD). These data were evaluated by one-way analysis of variance (ANOVA) using GraphPad Prism software (GraphPad Software, Inc., San Diego, CA, U.S.A.). *P*<0.05 was considered statistically significant.

## Results

### Low USP52 predicts a poor prognosis for NSCLC patients

**Figure 1:**
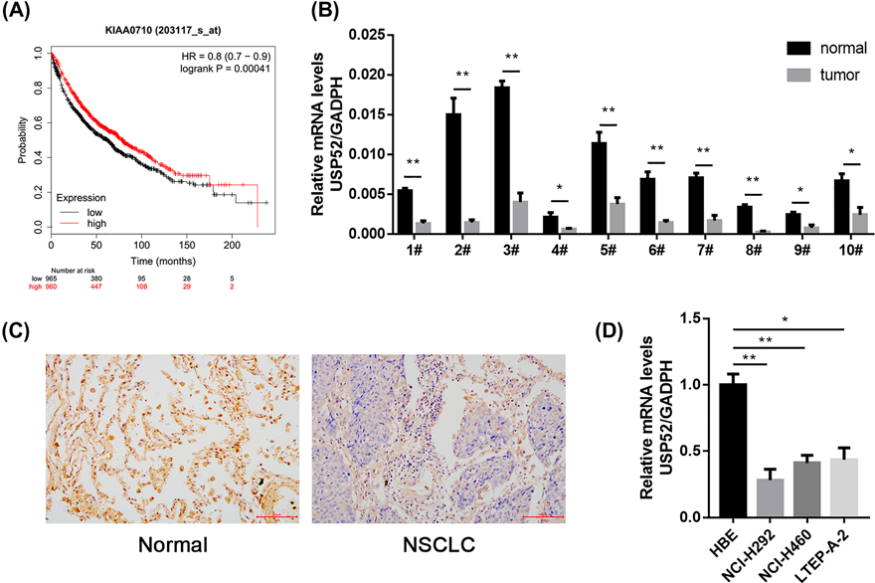
Low expression of USP52 predicts a poor prognosis for NSCLC patients (**A**) The overall survival periods of lung cancer patients with low or high expression of USP52 were estimated by Kaplan–Meier plotter (http://kmplot.com). (**B**) Ten pairs of fresh primary NSCLC tumor tissues (T) and individual normal paracancerous tissues (N) were prepared for qRT-PCR against USP52. (**C**) Representative images of the IHC staining for USP52 in NSCLC tissues and normal paracancerous tissues. (**D**) Three NSCLC cell lines and one HBE were prepared for qRT-PCR against USP52. *P<0.05, **P<0.01.

Deubiquitination plays a decisive role in lung cancer, such as USP7 [5]. USP52 acts as a deubiquitinase and promotes histone chaperone ASF1A stabilization [8]. However, the function of USP52 in NSCLC has not been reported. In order to explore it, we screened publicly available datasets to determine the prognostic correlation between the USP52 level and survival of NSCLC patients. Kaplan–Meier survival analysis revealed that the survival time for patients with low USP52 expression was obviously shorter than those with high USP52 expression (*P*=0.00041) (Figure 1A).

We then examined the mRNA expression of USP52 in ten pairs of fresh primary NSCLC tissues compared with individual normal paracancerous tissues. The result showed that, the mRNA expression levels of USP52 were dramatically reduced in NSCLC tissues compared with normal tissues (Figure 1B). Consistently, IHC staining assays further confirmed that, the expression of USP52 protein was also dramatically reduced in the NSCLC tissues compared with normal tissues (Figure 1C). Moreover, the expression of USP52 was also detected in human NSCLC cell lines (H292, NCI-H460 and LTEP-A-2). Consistent with the human specimen, the result show that USP52 were also significantly low expression compared with the HBE (Figure 1D).

Collectively, it manifested that low level of USP52 expression was associated with poor prognosis for NSCLC patients.

### Overexpression of USP52 inhibits cell growth in NSCLC cells

The above data revealed that the USP52 is lowly expressed in NSCLC, and we speculated that USP52 is a tumor suppressor gene in NSCLC. In order to study whether USP52 inhibits the proliferation of NSCLC tumor cells, we took the following research.

**Figure 2:**
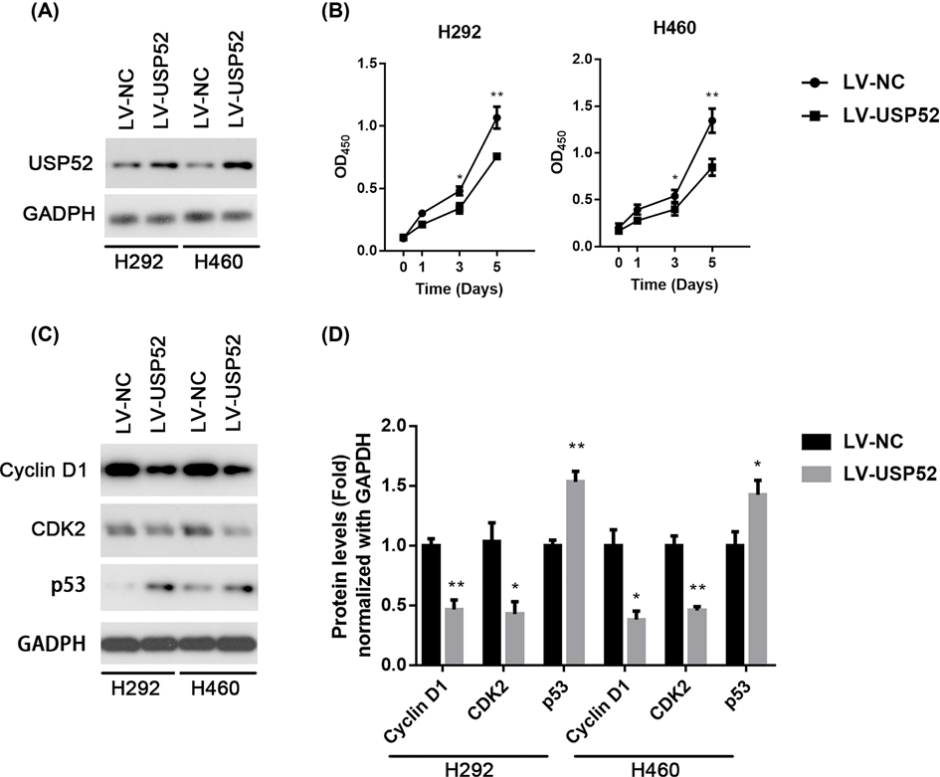
Overexpression of USP52 inhibits cell growth in NSCLC cells (**A**) H292 and H460 cells were stably infected with lentiviral LV-NC or LV-*USP52*, followed by Western blot against USP52. GAPDH was used as a loading control. (**B**) H292 and H460 cells stably infected with LV-NC or LV-*USP52* were cultured for indicated time, followed by CCK-8 assay at days 0, 1, 3 or 5. (**C**) H292 and H460 cells were stably infected with l LV-NC or LV-*USP52*, followed by Western blot against CCND1, CDK2, p53 and GAPDH. (**D**) The relative abundance of CCND1, CDK2 and p53 in H292 and H460 from (C) were quantified by gray scanning after LV-*USP52* plasmids transformed at 0, 1, 2, 4 μg. These experiments were repeated for three times. One-way ANOVA, *P<0.05, **P<0.01. NS, not significant.

Firstly, we established H292 and H460 cell lines with overexpression of USP52. Overexpression of USP52 was successfully verified in protein expression (Figure 2A). Then the CCK-8 analysis was used to analyze the cell proliferation at 0, 1, 3 and 5 days after overexpression of USP52. The OD value measured at 450 nm showed that the proliferation of the H292 and H460 cells were significantly inhibited compared with the control group after USP52 overexpression (*P*<0.05) (Figure 2B). It proves that USP52 suppresses NSCLC by inhibiting cell proliferation.

Further, we wanted to demonstrate the mechanism of how USP52 inhibits the proliferation of NSCLC. It was well known that CCND1 regulates cell proliferation [18]. CCND1 is believed to promote NSCLC cell proliferation and functions as regulators for CDKs [19]. The cell cycle is a tightly regulated process controlled by CDK–cyclin complex [20]. CDK2 was also known to promote cell proliferation [21]. In this study, the expression of CCND1 and CDK2 were detected in H292 and H460 cells by overexpression of USP52. The result shows that the expression of CCND1 and CDK2 were decreased after USP52 overexpression. Conversely, the expression of tumor suppressor p53 was increased (Figure 2C,D).

Together, these results indicated that the overexpression of USP52 could inhibit cell proliferation of NSCLC, and it was declared that USP52 suppresses NSCLC through the mechanism of inhibiting cell cycle regulatory proteins of CCND1 and CDK2, as well as promoting p53 expression.

### USP52 inhibits AKT signaling in NSCLC cells

**Figure 3:**
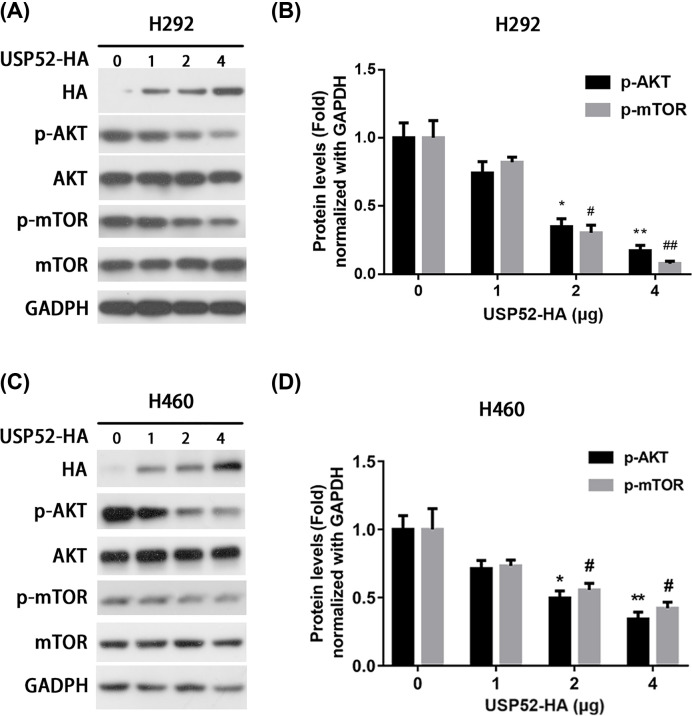
USP52 inhibits AKT signaling in NSCLC cells (**A**) H292 cells were transfected with empty vector (EV) or USP52-HA plasmids for 48 h, followed by Western blot against p-AKT, AKT, p-mTOR, mTOR and HA. GAPDH was used as a loading control. (**B**) The relative abundance of p-AKT and p-mTOR in H292 from (A) were quantified by gray scanning after USP52-HA plasmids transformed at 0, 1, 2, 4 μg. (**C**) H460 cells were transfected with empty vector (EV) or USP52-HA plasmids for 48 h, followed by Western blot against p-AKT, AKT, p-mTOR, mTOR and HA. GAPDH was used as a loading control. (**D**) The relative abundance of p-AKT and p-mTOR in H460 from (C) were quantified by gray scanning after USP52-HA plasmids transformed at 0, 1, 2, 4 μg. These experiments were repeated for three times. Groupt p-AKT: One-way ANOVA, *P<0.05, **P<0.01. Group p-mTOR: One-way ANOVA, ^#^*P*<0.05, ^##^*P*<0.01. NS, not significant.

Same as CCND1 and CDK2, it has been reported that AKT/mTOR pathway also plays crucial role in promoting cell growth [22]. In order to further understand whether USP52 decreases cell growth by regulating AKT/mTOR signaling, H292 and H460 cells were transfected with dose-dependent USP52 plasmid. In response to USP52 overexpression, the level of phospho-AKT (p-AKT) and phospho-mTOR (p-mTOR) was markedly reduced (Figure 3A–D), which verifies the activation status of AKT/mTOR pathway. These results indicated that USP52 suppresses NSCLC via inhibiting the activation of AKT/mTOR pathway.

### USP52 and PTEN were decreased in NSCLC tissues

**Figure 4:**
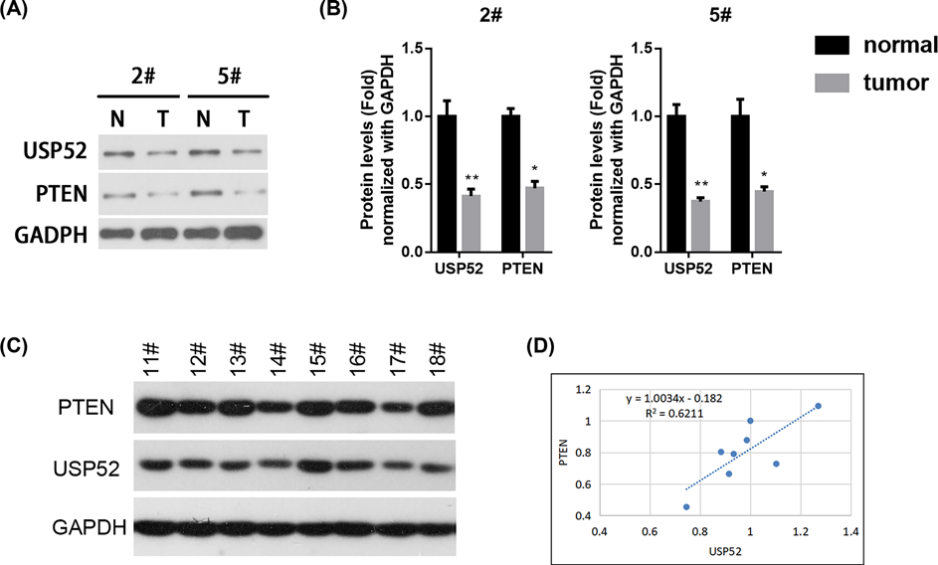
USP52 and PTEN were decreased in NSCLC tissues (**A**) Two representative pairs of fresh primary NSCLC tumor tissues (T) and individual normal paracancerous tissues (N) were prepared for Western blot against USP52 and PTEN. (**B**) The relative abundance of USP52 and PTEN in N and T from (A) were quantified by gray scanning as 2# and 5# patients, respectively. These experiments were repeated for three times. One-way ANOVA test, *P<0.05, **P<0.01. NS, not significant. (**C**) Eight representative pairs of fresh primary NSCLC tumor tissues were prepared for Western blot against USP52, PTEN and GAPDH. (**D**) Linear function was used to analyze the correlation between USP52 and PTEN expression, and fit linear function Y = 1.0034x-0.182, R^2^ = 0.6211.

PTEN inhibits the PI3K/AKT pathway by catalyzing the dephosphorylation of PIP3, while the loss of PTEN induces the activation of PI3K/AKT cascade, thereby stimulating cell growth and survival [23]. To confirm the correlation between PTEN and USP52 in lung cancer, firstly, we quantified the expression of PTEN and USP52 in lung cancer tissues. Western blot results further revealed that PTEN and USP52 expression were also notably decreased compared with normal paracancerous tissues (Figure 4A,B). Further experiments found that the expression levels of PTEN and USP52 in tumor tissues have a certain correlation (R^2^ = 0.6211, Figure 4C,D).

We hypothesized that the decrease in PTEN was on account of the low expression of USP52, that is, USP52 inhibited the tumorigenesis of NSCLC by up-regulating the tumor suppressor of PTEN.

### USP52 stabilizes PTEN protein in NSCLC cells

**Figure 5:**
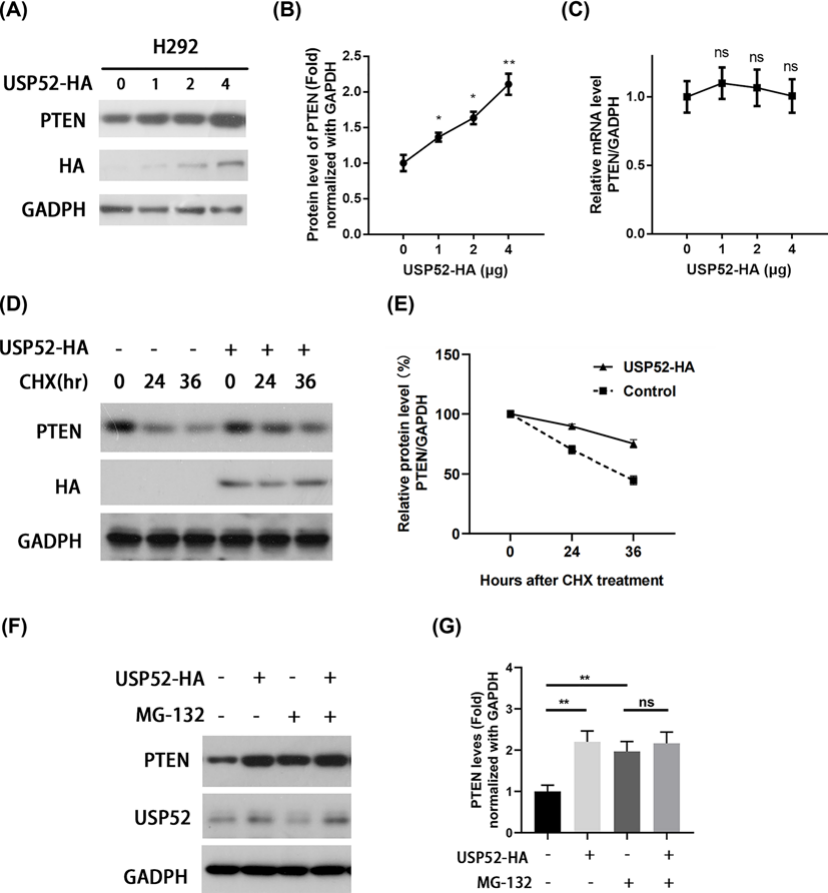
USP52 stabilizes PTEN protein in NSCLC cells (**A**) Control or USP52-HA plasmids were transfected into H292 cells for 48 h, followed by Western blot against PTEN and HA. (**B**) The relative abundance of PTEN in H292 from (A) was quantified by gray scanning after USP52-HA transforming at 0, 1, 2, 4 μg. These experiments were repeated for four times. One-way ANOVA, *P<0.05, **P<0.01. NS, not significant. (**C**) qRT-PCR against PTEN after USP52-HA transformed at 0, 1, 2, 4 μg. (**D**) H292 cells were transfected with USP52-HA or empty vector (Control) for 24 h, followed by CHX chase assay at indicated time. Then, Western blot was performed to assess the expression levels of PTEN and USP52-HA. (**E**) Statistical analysis of (D). (**F**) H292 cells were transfected with USP52-HA plasmid (control) and treated with MG-132 respectively, and then Western blot was performed on PTEN, USP52 and GADPH, where - means untreated (control). (**G**) The relative abundance of PTEN, USP52 and GADPH in H292 from (F) was analyzed by quantitative statistical analysis, **P<0.01. NS, not significant.

It has been reported that CK1α could suppress lung tumor growth by stabilizing PTEN [24]. In order to confirm the regulatory relationship between USP52 and PTEN, we analyzed the expression of PTEN in USP52 overexpressed H292 cells. Western blot and qPCR have been used to test the protein and mRNA, respectively. Western blot results exhibited that the expression of PTEN protein in USP52 overexpressed H292 cell lines was significantly higher than that in control group in a dose-dependent manner (Figure 5A,B). Whereas the expression levels of PTEN mRNA in USP52 overexpressed H292 cell lines has no significant difference compared with the control group (Figure 5C). It is suggested that USP52 may regulate the expression of PTEN through a post-transcriptional mechanism.

Furthermore, we determined to explore whether USP52 affects PTEN protein stability. Here, we used the protein synthesis inhibitor CHX to inhibit the transport step in protein synthesis (the movement of mRNA and two tRNA molecules on the ribosome) and block the extension of peptides during translation, resulting in cell growth arrest even death. Indeed, PTEN was remarkably attenuated and degraded slower in H292 cells after USP52 overexpression (Figure 5D,E). When the proteasome pathway is inhibited by MG-132, the ability of USP52 to regulate PTEN is inhibited, which further proves that USP52 affects the protein level of PTEN through the deubiquitination pathway (Figure 5F,G). In general, the above results imply that the USP52 inhibit NSCLC cell lines proliferation through the mechanism of strongly promotes the protein stability of antioncogene PTEN.

## Discussion

Ubiquitination regulation is widely involved in the regulation of various biological processes including autophagy [25], cell-cycle progression, signal transduction, transcriptional regulation, receptor down-regulation and endocytosis [26]. Abnormalities in ubiquitin-mediated processes have been shown to cause pathological conditions including malignant transformation [26]. Although USP52 exhibits no deubiquitylating activity on account of the lack of an active-site cysteine residue [27], it was required for *HIF1A* mRNA stability [6]. Recently, it has been reported that USP52 could promotes histone chaperone ASF1A stabilization [8]. In this study, we have found that USP52 was expressed at a low level in NSCLC specimen in NSCLC specimen, and the survival time for patients with low USP52 expression was obviously shorter than those with high USP52 expression. This indicated that USP52 play an important role in NSCLC suppression.

Tumor-related cell cycle defects are usually mediated by alterations in the activity of CDK. Dysregulated CDKs induce unscheduled proliferation as well as genomic and chromosomal instability [28]. Emerging evidence suggests that tumor cells may also require specific interphase CDKs for proliferation [28]. The overexpression of CCND1, a cell cycle-regulating protein, is associated with poor histological differentiation in NSCLC [11,29]. In this study, we have found that USP52 overexpression in NSCLC cell lines could inhibit CCND1 and CDK2 expression, which suggested that USP52 suppresses NSCLC through the mechanism of inhibits the expression of cell cycle regulatory proteins of CCND1 and CDK2.

The gene encoding the components of the PI3K-AKT-mTOR signaling axis is often mutated in cancer, but mTOR, which encodes mTOR kinase, is rarely mutated. The researchers used publicly available tumor genome sequencing data identifying 33 mTOR mutations that confer pathway hyperactivation [30]. In this research, we have identified that USP52 overexpression inhibits the activation of AKT/mTOR pathway, which suggested that USP52 also inhibits AKT/mTOR pathway to suppress NSCLC. Whereas the mechanism remains to be further explored.

The anticancer activity of PTEN mainly depends on its lipid phosphatase activity, which is contrary to the activation of PI3K/AKT. As a typical tumor suppressor gene that inhibits the cascade of PI3K/AKT/mTOR pro-growth signals, PTEN dysfunction leads to misadjustment of this pathway and other pathways, leading to excessive growth [23]. PTEN regulates many cellular processes, including proliferation, survival, energy metabolism, cell structure and movement. More than a decade of research has expanded our knowledge of how PTEN is controlled at the transcriptional level and regulates its enzyme activity, protein stability and cell location through extensive post-transcriptional modifications [31]. In this study, we found that the overexpression of USP52 could up-regulate PTEN. In addition, USP52 strongly stabilizes PTEN protein in NSCLC cells.

In conclusion, the present study demonstrated that USP52 is essential for inhibiting the NSCLC cells proliferation. The low expression of USP52 predicts a poor prognosis for NSCLC patients. And indicated that USP52 suppresses the NSCLC cells proliferation through the mechanism of inhibiting the cell cycle regulator of CCND1, CDK2 and AKT/mTOR pathway. Furthermore, USP52 suppresses the NSCLC cells proliferation through the mechanism of promoting the protein stability of PTEN via the deubiquitination.

## Data Availability

All supporting data are included within the main article.

## Abbreviations

CCK-8: Cell Counting Kit-8
CCND1: cyclin D1
CDK: cyclin-dependent kinase
CHX: cycloheximide
FBS: fetal bovine serum
GAPDH: Glyceraldehyde-3-Phosphate Dehydrogenase
HBE: human bronchial epithelial cell
HIF1α: Hypoxia Inducible Factor 1 Subunit Alpha
IHC: immunohistochemistry
NSCLC: non-small cell lung cancer
PI3K: phosphatidylinositol 3-kinase
PTEN: phosphatase and tensin homolog
p-AKT: phospho-AKT
p-mTOR: phospho-mTOR
USP: ubiquitin-specific peptidase
